# Towards High Capacity Li-ion Batteries Based on Silicon-Graphene Composite Anodes and Sub-micron V-doped LiFePO_4_ Cathodes

**DOI:** 10.1038/srep37787

**Published:** 2016-11-29

**Authors:** M. J. Loveridge, M. J. Lain, I. D. Johnson, A. Roberts, S. D. Beattie, R. Dashwood, J. A. Darr, R. Bhagat

**Affiliations:** 1 Warwick University, Coventry, CV4 7AL, UK; 2 University College, London, WC1E 6BT, UK.; 3 Coventry University, Coventry, CV1 5FB, UK.

## Abstract

Lithium iron phosphate, LiFePO_4_ (LFP) has demonstrated promising performance as a cathode material in lithium ion batteries (LIBs), by overcoming the rate performance issues from limited electronic conductivity. Nano-sized vanadium-doped LFP (V-LFP) was synthesized using a continuous hydrothermal process using supercritical water as a reagent. The atomic % of dopant determined the particle shape. 5 at. % gave mixed plate and rod-like morphology, showing optimal electrochemical performance and good rate properties vs. Li. Specific capacities of >160 mAh g^−1^ were achieved. In order to increase the capacity of a full cell, V-LFP was cycled against an inexpensive micron-sized metallurgical grade Si-containing anode. This electrode was capable of reversible capacities of approximately 2000 mAh g^−1^ for over ^1^50 cycles vs. Li, with improved performance resulting from the incorporation of few layer graphene (FLG) to enhance conductivity, tensile behaviour and thus, the composite stability. The cathode material synthesis and electrode formulation are scalable, inexpensive and are suitable for the fabrication of larger format cells suited to grid and transport applications.

Energy storage demands for next generation electric vehicles and grid storage have increased significantly during the last decade, with lithium ion technology remaining the most likely contender to meet these requirements in the short to medium term. The key requirements for vehicle energy storage are high gravimetric and volumetric energy density, whereas for grid storage, cycle life is the most important parameter. To obtain high energy density batteries, silicon anode materials with a theoretical capacity of 3579 mAh g^−1^ have been a major focus of recent battery research to replace graphite (with a limited capacity of only 372 mAh g^−1^)^1^,^2^,^3^,^4^,^5^,^6^. There are still performance issues surrounding silicon that have prevented its successful commercialization to date. These include significant volume expansion upon lithiation (280 vol %), irreversible capacity loss due to solid-electrolyte interphase (SEI) growth during cycling and lithium isolation within particles^7^. To enhance the performance of the silicon anode, this study uses micron-sized crystalline particles (compared with the much-developed nano structures) and a hierarchy of conductive carbons, including FLG. This offers a more continuous, effective network to maximize electronic contact between active material particles and the copper current collector, and can generate a mechanically stable composite architecture. A key consideration is the uniform dispersion of the carbon additives in order to avoid inducing structural inhomogeneities that would result in unevenly distributed stress in the electrode during the silicon volume changes. This can degrade the electronic connections between the active material and the conductive-binder network, the electrode’s so-called “wiring”.

In terms of advancing cathode material performance, olivine lithium iron phosphate (LFP) based nanomaterials have demonstrated superior power (rate) performance, thermal stability, cycle life and are considered relatively environmentally benign, compared with other known Li-insertion compounds such as manganese-spinel^8^. Other attractive properties of LFP include reduced reactivity with industry-standard electrolytes and a flat charge-discharge curve during cycling. This is due to a two-phase α ↔ β transition that occurs during Li-extraction/insertion processes^9^ resulting in relatively high capacities of *ca*. 170 mAh g^−1^. Despite these advantages, initial concerns for these materials related to rate performance, where capacity drop was significant when using high current densities. To overcome these issues, research has focused on nano-sizing and carbon coating LFP, which has improved the kinetics of electron transfer and reduced the limitations from slow Li-ion diffusion^7^. In addition, doping of vanadium (or other ions) within the LFP structure, has been reported to improve the discharge capacity at high rates by numerous researchers^10^,^11^,^12^,^13^,^14^. In the case of V-doped LFP, the improved performance has been attributed to increased electronic conductivity, decreased diffusion activation energy barriers and increased solid-solution miscibility with Li within the material^10^,^11^. In some cases, conductive vanadium-containing impurity phases (such as Li_3_V_2_(PO_4_)_3_ or VO_2_) on the LFP surface, have also been partially attributed to the improved performance by increasing electron and Li^+^ transport dynamics across the surface^12^,^13^,^14^.

Continuous hydrothermal flow synthesis (CHFS) is a relatively underexplored method for the production of battery anodes^15^,^16^,^17^ and cathodes^18^ including V-LFP nanomaterials. The process involves the reaction of a feed of supercritical water with a reagent stream in an engineered mixer, over a period of a few seconds or minutes. This results in the formation of a metal oxide or phosphate material, usually of small particle size due to the supersaturated mixing conditions of the synthesis^19^. The CHFS process is scalable as reported by some authors^20^,^21^, where production of ZnO has been successfully scaled up by a factor of 40 with little change observed in the crystallite size^21^. This process can be used to make crystalline nanomaterials rapidly with controlled particle properties compared with more common synthesis methods, such as traditional solid-state (polydispersed grain growth due to the high processing temperatures) or batch hydrothermal approaches^18^,^21^,^22^. A major benefit of the solution route is that nucleation and growth phenomena can be controlled more easily to generate materials with desirable phase purity, grain size and morphology. Another benefit is that a uniform carbon coating is easily achieved and is a widely accepted method for improving the electronic properties^23^. The lithium ion conductivity was experimentally shown to occur predominantly along the a- axis and was negligible along the b- and c-axes^9^,^24^.

Graphene has attracted a great deal of publicity and scientific curiosity since its discovery in 2003. Furthermore, different types and grades of graphene are becoming increasingly available, at cost competitive prices. Modification of the LFP surface with graphene has been shown to deliver capacities of up to 208 mAh g^−1^, an increase of approximately 18% compared with the theoretical maximum of unmodified LFP (170 mAh g^−1^)^25^. This is said to occur via a reversible redox reaction between the Li ions of the electrolyte and the flake graphene coating. Similarly, a recent first principles study on the lithiation of a silicon-graphene composite, showed a significant enhancement of lithium ion mobility along the silicon-graphene interface^26^. Beyond reducing and controlling the particle size and degree of polydispersity, all of the major developments to enhance the high rate performance of LiFePO_4_ electrodes, have historically been the result of electrode-level improvements^27^. Such improvements seek to optimize the electrodes short and long-range conductive networks and ultimately homogenize the distribution of the applied electrochemical potential across the electrodes. Herein, the study focuses on nano-sized V-doped LFP material (as a cathode) along with an improved silicon anode, incorporating functionalised graphene nano-platelets (electrical conductivity X, Y = 107 S m^−1^ and Z = 102 S m^−1^) to enhance the conductivity and mechanical properties.

## Experimental

### Continuous Hydrothermal Flow Synthesis of V-doped LiFePO_4_

**Figure 1:**
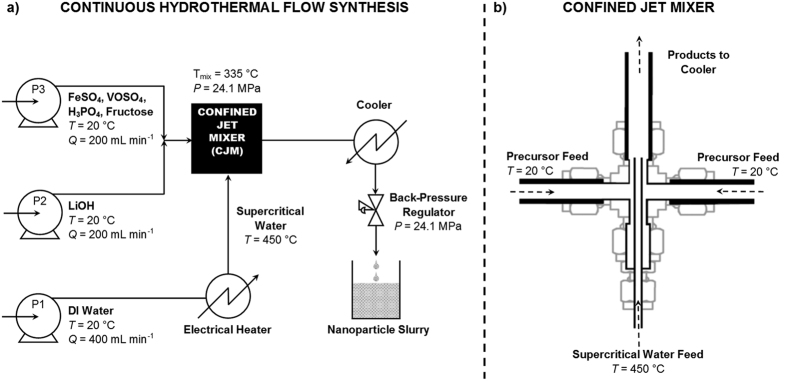
(**a**) Schematic diagrams of the continuous hydrothermal flow synthesis reactor system. FeSO_4_ = Iron sulphate, VOSO_4_ = vanadium oxide sulphate hydrate and H_3_PO_4_ = phosphoric acid; P = pump; LiOH = Lithium hydroxide. (**b**) Outlines the mixing head, the central component of the apparatus where the reagents are combined.

Carbon-coated vanadium-doped lithium iron phosphate (where the carbon is amorphous) was synthesized using a pilot scale continuous hydrothermal flow synthesis (CHFS) reactor at a rate of 0.25 kg h^−1^ in a similar manner to that previously reported^14^. Two aqueous solutions were prepared from the following precursors. 0.2375 M FeSO_4_·7H_2_O (99%, Alfa Aesar), 12.5 mM VOSO_4_·5H_2_O (17–23% V, Acros Organics), 0.375 M H_3_PO_4_ (85–88 wt %, Sigma Aldrich) and 0.65 M fructose (99%, Alfa Aesar) were combined for the first solution. The second solution was 0.8625 M LiOH·H_2_O (99%, Fischer Scientific). Both solutions were pumped into a T-piece mixer (6.35 mm internal diameter) each with a 200 mL min^−1^ flow rate, as shown in Fig. 1. The mixture of both solutions flowed at 400 mL min^−1^ into the side arms of a combined jet mixer (CJM), the design of which is described elsewhere^24^. In this region the solution rapidly combined with a turbulent jet of supercritical deionized water (450 °C and 24.1 MPa at a flow rate of 400 mL min^−1^), emerging from the inner tube of the CJM. The nanoparticles of LiFe_0_._95_V_0_._05_PO_4_/C formed in the mixture at a reaction temperature of *ca*. 335 °C. The mixture flowed through an outlet pipe at this temperature with a residence time of *ca*. 6.5 s (an effective measure of the reaction time) before cooling to near ambient temperature by a pipe-in-pipe counter-current heat exchanger. The cooled slurry flowed through a back-pressure regulator (BPR, Swagelok KHB series) which maintained a system pressure of 24.1 MPa, after which it was collected.

The particles were recovered by centrifugation and washed with deionised water. The observed yield was 0.50 kg from 90 L of product suspension. The wet powder was freeze-dried and then subsequently heat-treated at 700 °C for 3 hours (under flowing argon) to graphitize the carbon coating on the surface of the particles. The carbon content of the carbon-coated V-LFP was calculated to be 6.7 wt % C from carbon-hydrogen-nitrogen (CHN) analysis. The material was ball-milled for 1 h at 400 rpm using a Retsch planetary ball mill PM-200 using a 1:1 w/w ratio of LFP and N-methyl pyrrolidone (NMP) with 4 mm zirconia balls. The particle size distribution as a result reduced from a D_90_ particle size of 650 μm to 22 μm.

### Composite Anode Formulation

Silicon electrodes were prepared in multiple steps as outlined below. The composite electrodes were based on a combination of Si (purity > 99%, Elkem Bremanger): PAA polymer (Sigma Aldrich, MWT = 450 k, purity ≥ 99.5%) and conductive additives acetylene black (Alfa Aesar, purity 99.9%, S.A. 75 m^2^ g^−1^ and FLG (XG Sciences M Grade, purity > 99.9%, specific surface area specified in the range 120–150 m^2^ g^−1^).

1. Firstly, a solution of polyacrylic Acid (PAA; Sigma Aldrich, MWT = 450 k, purity ≥ 99.5%) was prepared by mixing 24 g of PAA with 176 g of deionised water (equates to 12 w/w % PAA), in a 500 mL Nalgene^®^ beaker. The PAA slurry was then mixed using a Primix Homodisper (Model 2.5) at 500 rpm for 120 minutes, followed by stirring at 250 rpm for a further 120 minutes with a Primix medium shear impeller blade, until the solution is clear and devoid of air bubbles. 12.4 g of sodium carbonate (Na_2_CO_3_; Fisher Chemical, purity > 99.5%) was added to the stock PAA solution. This results in a partial Na neutralization of the PAA carboxyl groups, which extends the polymer configuration and optimises interaction with conductive additives and active material. The mixture was stirred by hand, with a spatula, until all of the Na_2_CO_3_ was dissolved. The partially neutralized Na-PAA solution was left overnight until a clear solution resulted.
2. A conductive additive mixture was formulated using 10.0 g FLG, 5.0 g acetylene black, 136.4 g deionised water and 1.0 g of 12 w/w % PAA solution to give a C loading of 11.7 wt %. This suspension was stirred at 500 rpm using a Primix Homodisperser (Model 2.5), followed by static ultrasonication using a Hielscher sonic probe (Model UP400S) using 0.5 cycles and an amplitude of 60% for two 7 min sonication steps.
3. 20.0 g of Si powder was mixed with 91.5 g of C-mix and stirred using a Primix Homodisperser for 30 min at 100 rpm. 20.0 grams of Si (Elkem Silgrain e-Si, d_50_ 3.1 μm, purity 99.7%) was combined with the carbon solution and stirred for one hour (1000 rpm for 30 minutes followed by 500 rpm for 30 minutes) using a Primix high-shear impeller. The Si composite slurry was then subjected to ultrasonication to break down any Si/C agglomerates. 33.3 g of partially neutralized Na-PAA solution (described above) was added to all of the Si slurry described above. The composite slurry was further dispersed using a Primix Homodisper Model 2.5 for 30 minutes. The resulting solution was transferred to a Filmix mixing vessel in 30 mL aliquots and subjected to the following mix cycle: two dispersions for 30 s each at 10 m s^−1^ then 30 s at 25 m s^−1^.

Following degassing of the solution, anode coatings were cast onto 10 μm thick Cu foil (Oak Mitsui, electrodeposited), using a laboratory scale RK Instruments K Coating Proofer machine with a micrometer-assisted doctor blade coated. Electrodes were dried on a hot plate at 80 °C, followed by vacuum drying (7 mBar) for 12 hours at 70 °C. The above formulation resulted in electrodes with a dry mass % composition of 70:14:16 (Silicon: Na-PAA: carbon additives).

### Cathode Formulation

A cathode formulation of 80:10:10 wt % (V-LFP: PVdF: CB) was generated by mixing the V-LiFePO_4_ with carbon black (Timcal C65, Purity 99.9%, specific surface area 65 m^2^ g^−1^) and NMP (Sigma Aldrich). It is important to note that 6.64 wt% of the V-LFP material was carbon from the sucrose carbonization process, occurring from heat treatment of the V-LFP. The cathode was processed using the following steps:

1. A solution of polyvinylidene difluoride (PVdF) grade 5130 (Solvay) was formulated by dissolving 80 g PVdF powder in 920 g NMP. This was performed using a T2F Turbula mixing apparatus (WAB, Germany) for 12 hours until the PVdF is completely dissolved to produce a binder concentration of 8 wt %.
2. 144 g V-LFP and 16.6 g acetylene black were dry mixed in a HIVIS high torque mixer at 10 rpm for 10 min.
3. 208.1 g of the 8 wt % PVdF 5130 solution was added and the slurry was mixed for 30 min at 15 rpm.
4. 50 g of NMP was added to reduce the viscosity of the solution, with further mixing for 35 min at 15 rpm followed by 30 min at 100 rpm.
5. 70 g NMP was added prior to the final stage of high torque mixing under static vacuum for 90 min at 100 rpm.
6. The contents were transferred to a FilmixTM Model 56–50 Disperser for 0.5 mins at a lineal speed of 8 m s^−1^. The resulting solid content of the electrode formulation was 35 wt %.

Cathode coating on Al foil was carried out on a Reel-to-reel Coater (MEGTEC) using a comma bar set an incrementally increasing blade gaps in the range 50–240 μm to produce areal coating densities in the range 24–95 g m^−2^. The coating speed was fixed at 0.75 m min^−1^ using temperatures in three successive drying zones of 100, 120 and 110 °C respectively.

The graphene-containing cathode was made in a smaller-scale formulation using an 80:10:5:5 wt % ratio (V-LFP: PVdF: CB: FLG), and was processed using the following steps:

1. 20 g V-LFP, 1.25 g acetylene black and 1.25 g FLG were combined with 31.25 g of the 8 wt % PVdF 5130 solution and stirred with a Primix Homodisper Model 2.5 for 30 min, whilst continually adding 15.69 g of NMP to give a solid content of 36 wt %.
2. The mixture was transferred to a FilmixTM Thin-film Disperser Model 40–60 and dispersed at a lineal speed of 5 m/s for 0.5 min, 10 m s^−1^ for 0.5 min and 15 m s^−1^ for 15 sec.

Cathode coating on Al foil was carried out on using a draw-down coater (RK Instruments) to incrementally increasing blade gaps of 50–200 μm.

### Coin Cell Preparation and Electrochemical Characterisation

Coin cells for Si vs. Li/Li^+^ half-cells incorporated a Celgard separator (2325 grade) which is a porous polyolefin film. The electrolyte used was EC: EMC (3:7), with 15 wt % FEC and 3 wt % VC. The cycling voltage range for Si electrodes in a half cell configuration was 0.005 to 1.0 V. The first (formation) cycle used a relatively low current (±C/25), followed by higher currents on subsequent cycles (±C/5). For some tests, the lithiation step was limited by capacity rather than voltage. In these tests, the capacity limit on the first cycle was higher than on subsequent cycles. Differential plots of dQ/dV were calculated directly from the data.

Half-cell tests for the V-LFP were performed on two electrode 2032-type coin cells, which were assembled in an argon-filled glovebox (MBraun UNIlab) with O_2_ and H_2_O maintained below 10 ppm. The counter electrode was lithium metal foil (PI-KEM). The separator, a glass microfiber filter (WHATMAN), was saturated with an organic electrolyte (LiPF_6_ in 3:7 wt% ethylene carbonate/ethyl methyl carbonate, LP57 electrolyte (BASF). Electrochemical measurements were performed using an Arbin Instruments potentiostat at room temperature of 20 °C. Galvanostatic charge/discharge cycling tests (specific current tests) were performed in the range of 2 to 4.3 V vs. Li/Li^+^, applying variable specific currents in the range 0.05 and 9 A g^−1^ during charge and discharge. The specific current and specific capacity was calculated based on the mass of active material (i.e. V-LFP) in each electrode.

### Three-electrode Cells

**Figure 2:**
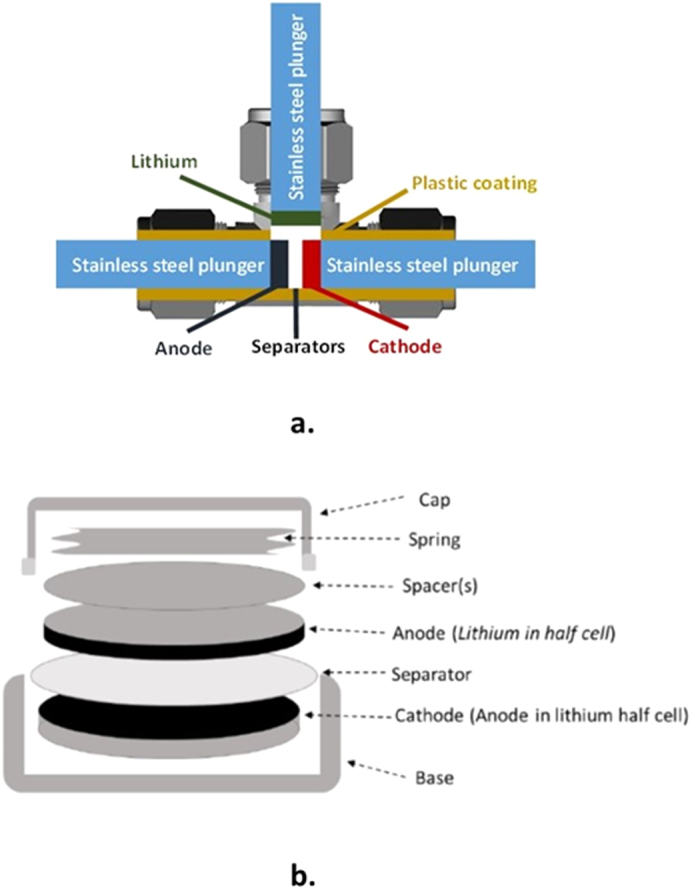
Electrochemical test cells used for evaluation of electrochemical performance of battery electrodes. (**a**) 3-electrode Swagelok cell with a reference Li electrode. (**b**) Schematic of the components within a 2032-type coin cell.

All cell components were dried in a vacuum oven at 50 °C overnight prior to assembly. Three-electrode cells were fabricated using stainless steel Swagelok^®^ hardware and perfluoroalkoxy (PFA) ferrules – as shown in Fig. 2. 60 μL of LP30 electrolyte was used (containing 1 M LiPF_6_), with EC: DMC 1:1, with 10% FEC and 5% VC. Connections to the three-electrode cell were made using connectors suited to a Biologic VMP3 potentiostat.

### Cycling procedure

The three-electrode cell was charged (silicon lithiation) at a constant C/20 rate followed by subsequent cycling at C/5. The charging was limited by a cathode voltage of 3.95 V vs. Li/Li^+^. Discharge (delithiation of silicon) was limited by an anode voltage of 1.50 V vs. Li/Li^+^. Coin cell characterisation was performed using a Maccor cycling unit, with all cells housed in Votsch VT-3050 environmental chambers maintained at 25 °C.

## Results and Discussion

### Material and Electrode Characterization

All microscopic and crystallinity characterisation is illustrated in Supporting Information Figures S1–S3. The heat-treated V-LFP was characterised using a JEOL Field Emission Transmission Electron Microscope (FE-TEM). From Figure S1 it can be seen that the carbon coating was smooth and uniform on the particle surface, although there was some evidence of small particles of pure carbon. X-ray powder diffraction data collection was performed using a Stoe & Cie GmbH XRD with 0.3 mm capillary, with Mo K-alpha radiation. FE-SEM (Field Emission Scanning Electron Microscopy) images were obtained using a JEOL JSM-6700F microscope, indicating the particle size. Figure S3 shows a mixed morphology of platelet-like and rod-like particles – the authors have previously reported that vanadium doping can influence the formation of platelet-like LFP particles within the CHFS process^18^ Peak fitting was carried out using the LeBail method^28^. Inspection of the XRD patterns from Figure S3 indicate that there are no impurities evident, with peaks characteristic for the expected orthorhombic pnma space group symmetry of olivine LFP. This is consistent with V-LFP synthesised by the authors previously, where V was found to reside on both Fe and P sites with oxidation states of 3^+^ and 5^+^ respectively from EXAFS (Extended X-ray Absorption Fine Structure) and DFT (Density Functional Theory) analysis^18^. This gave a systematic distortion of the unit cell with V content, decreasing the *b* parameter and increasing the *c* parameter^18^. The lattice constants of the sample in this study deduced from LeBail analysis were: *a* = 10.3278(2) Å, *b* = 6.0044(1) Å, *c* = 4.6964(1) Å (R_wp_ = 7.62).

Upon examination of the microstructure of the Si-FLG electrodes it can be seen in Figure S2b that the dispersion of the carbon additives was reasonably uniform, which would be expected to provide effective connectivity between the active materials and the additives down to the current collector. Beyond reducing and controlling the particle size and degree of polydispersity, all of the major developments to enhance the high rate performance of LFP electrodes have historically been the result of electrode-scale improvements^27^. Both electrode materials appear to be uniformly dispersed, reducing the likelihood of charging and discharging inhomogeneities resulting from poor ionic (Li^+^ availability) and electronic connectivity throughout the electrode microstructure.

### Half-Cell Electrochemical Properties

#### C/V-LFP vs. Li

**Figure 3:**
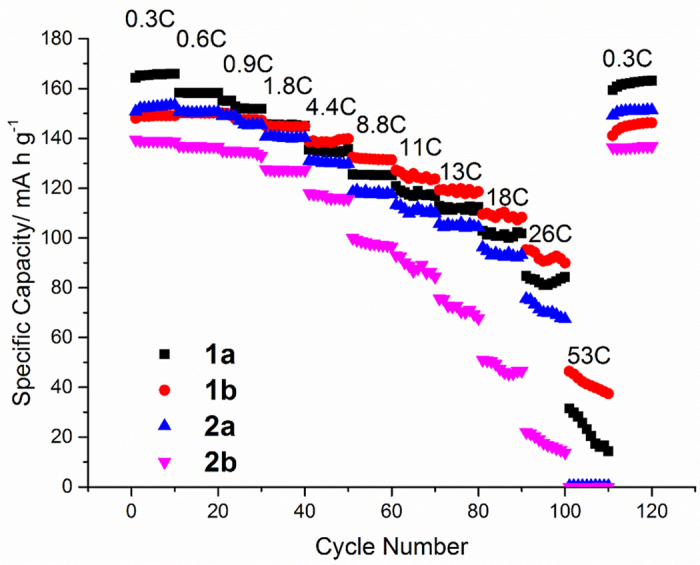
C rate vs. capacity for the V-LFP cathode vs. Li/Li^+^.

The electrochemical performance of the LFP and Si FLG electrodes were initially evaluated in half cells, to assess the reversible capacity and rate performance for anode and cathode materials. Figure 3 outlines the rate evaluation of the V-LFP electrode discharged at eleven increasing current densities, corresponding to C rates in the range 0.3 C to 53 C, where 1 C corresponds to a full discharge in one hour of a material assigned with a theoretical capacity of 165 mAh g^−1^. This value is slightly less than the value for pure undoped LFP (170 mAh g^−1^) because it only assumes the Fe and not the V is electrochemically active in this case. This is confirmed by Supporting Figure S5 showing voltage vs. capacity in a V-LFP half-cell (vs. Li) cycled at 0.9 C, where only the Fe^2+^/Fe^3+^ couple at 3.45 V is observed. Such high rates outlined in Fig. 3 were incorporated into the testing regime because rapid rate capability is one of the prominent advantages of the LFP family of materials. The plot compares two vanadium-doped LFP cathode formulations without graphene (electrodes 1a and 1b) and with graphene (electrodes 2a and 2b) and also the effect of increasing coating weight. As expected, the capacity decreased from ca. 165 mAh g^−1^ to < 40 mAh g^−1^ as charge and discharge rates of 50 C or higher were approached. At lower C-rates starting from the range 0.3 to 0.9 C, higher discharge capacities were demonstrated by the cathode with mass loading 2.6 g m^−2^ (electrode 1a). Conversely at C-rates in the range 1.8 C to 26 C, the graphene-containing electrodes with a mass loading of 2.4 g m^−2^ (electrode 2a) achieved the highest capacity of the electrode set. This may have been due to a slight decrease in electrode resistance. At high C-rates there was most likely to be diffusion-limited mass transfer of Li^+^ ions between the surface and the core of the C/V-LFP nanoparticles. For the electrodes with reduced mass loading, it was observed that decreasing the current from 53 C back to 0.3 C, a specific capacity of 160 mAh g^−1^, was recovered (97% of the initial discharge capacity). At each C rate, the storage capacity appeared to be more stable with the electrodes with lower mass loadings. Capacity loss at higher cycling rates may be attributed to overall electrode resistance, and the aforementioned limiting kinetics of Li^+^ diffusion in the V-LFP particles.

**Figure 4:**
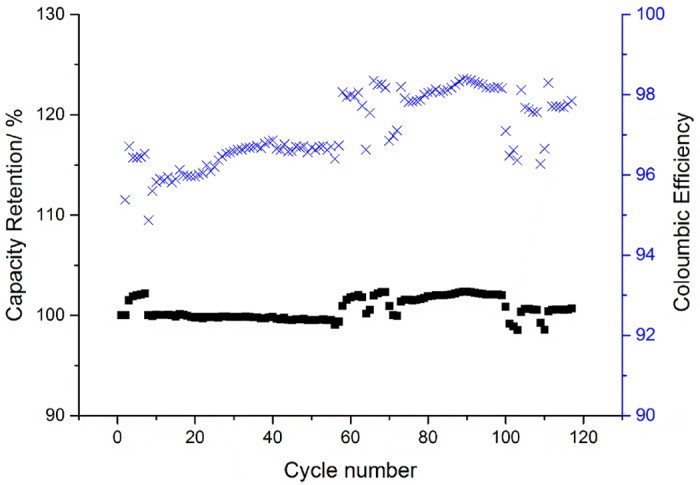
Capacity retention of V-LFP vs. Li/Li as a function of cycle number (blue data points represent the coulombic efficiency profile over the cell’s lifetime).

Coulombic Efficiency (CE) is defined as the ratio of the charge delivered during the discharge (Q_dis_) to that stored during charge (Q_ch_) and such measurements are useful to evaluate the impact of electrode active material chemistry and formulation. It can be seen from Fig. 4 that the CE for the LFP half-cell started at ca. 96%, and never increased above 99%, representing the rate of cathode capacity fade per cycle. There are several phenomena that can result in low CE of the LFP material and this is discussed later in the three-electrode electrochemical cell testing section.

#### Si vs. Li Half Cell

**Figure 5:**
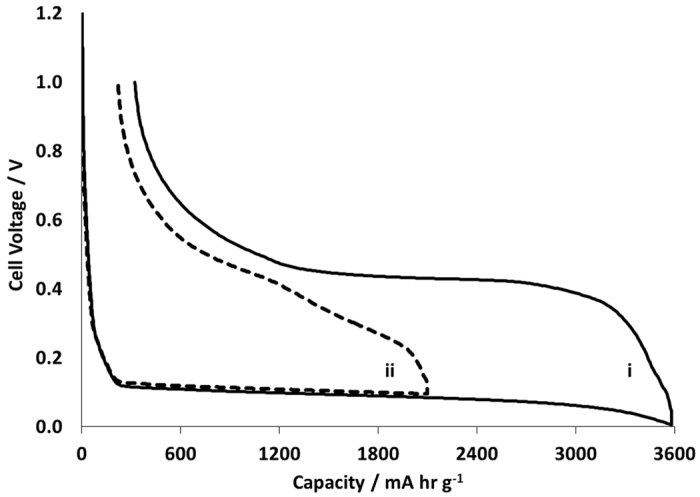
Voltage vs. capacity profiles for the first cycle of a Si vs. Li/Li^+^ cell charged to full and half capacity (profile i and ii respectively).

Figure 5 shows the voltage profiles for high (3579 mAh g^−1^) and medium (2099 mAh g^−1^) capacity silicon coin cells vs. Li/Li^+^. The shape of the profile and plateau positions are consistent with previous findings^29^. The low V plateau corresponds to a two-phase region in which lithiated amorphous silicon is formed^30^. The composition of a-Li_x_Si varies during the lithiation reactions and can only be considered pseudo-equilibrium. Lithiation-induced expansion has been reported to occur predominantly at the a-Si/a-Li_x_Si phase boundary and the [Li] gradient is negligibly small in the lithiated layer of a-Li_x_Si when x reaches ~2.5^30^. According to Yoon^31^ the lower voltage plateau observed has a capacity of around 3250 mAh g^−1^ when the crystalline Si has entirely reacted to form a-Li_x_Si. Where the V slopes further down to <50 mV, the Li_15_Si_4_ phase crystallizes within the lithiated Si.

**Figure 6:**
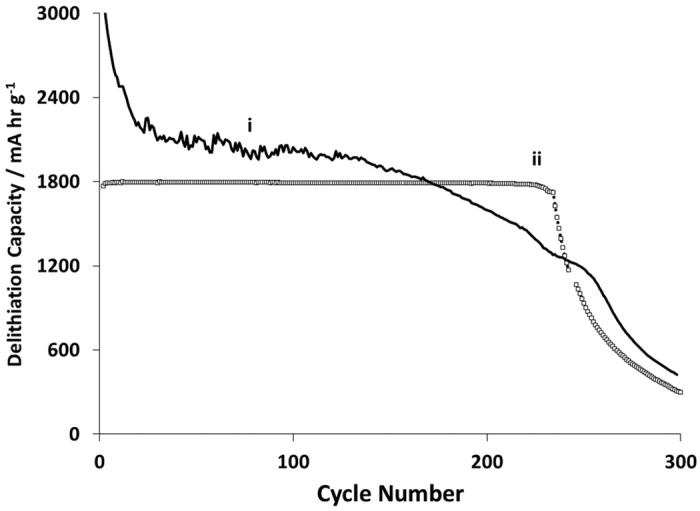
Delithiation capacity of the Si-FLG anode as a function of cycle number with anodes cycled at (i) full and (ii) half capacity.

The irreversible capacity loss on the first cycle (first cycle loss – FCL) was *ca*. 9% in (i) and 10.7% in (ii). Figure 6 shows the delithiation capacity as a function of cycle number in the range 2099 to 3583 mAh g^−1^, for 100 charge-discharge cycles. A steady decline in capacity can be seen, with the highest capacity cell falling to *ca*. 70% capacity retention after only 100 cycles. This is attributable to the electronic and/or ionic or mechanical isolation of silicon particles with observable decline in the coulombic efficiency of the cell.

### Three-electrode Electrochemical Performance

**Figure 7:**
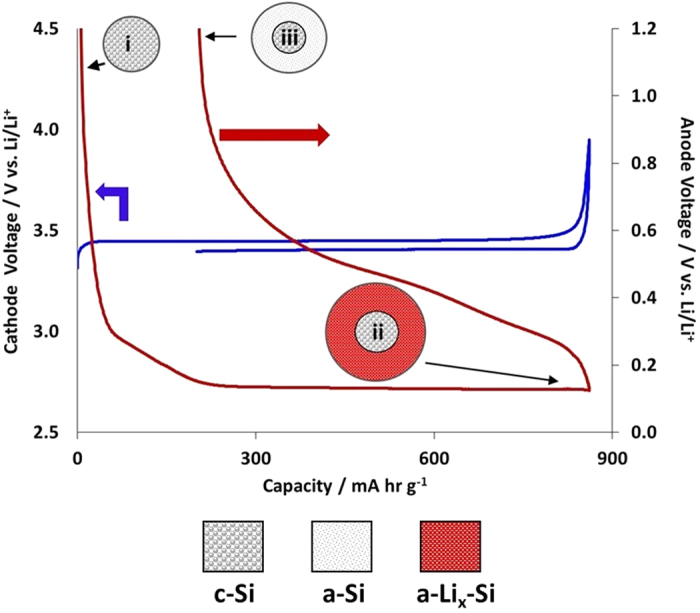
Voltage profiles on the first cycle for the Si and V-LFP electrodes with illustrated structural transformations when the Si undergoes the crystalline-to-amorphous transition during lithiation on the first cycle.

Figure 7 depicts the galvanostatic charge-discharge profiles for the first cycle of the Si-FLG anode and V-LFP cathode against a lithium reference electrode. The lithiation plateaus of the silicon electrode were consistent with the lithiation/delithiation events reported to form at certain voltages between 1.5 and 0.1 V. Due to the relative areal capacities, the silicon capacity did not exceed 1000 mAh g^−1^. Thus, none of the Li_15_Si_4_ phase was expected to form; this requires an electrode voltage below 0.05 V^29^. The capacity is quoted relative to the active mass of the silicon electrode. The V-LFP electrode was cycled in the range 2.5–4.0 V, and demonstrated single charge-discharge plateaus at ca. 3.45 and 3.39 V vs. Li/Li^+^, for charge and discharge, respectively. This confirmed the simple lithium extraction-insertion reaction in pure olivine LFP phase as shown by Equation 1.


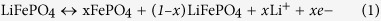


The electrochemical extraction of Li from LiFePO_4_ gives the Fe^2+^/Fe^3+^ redox potential at *ca*. 3.45 V vs Li/Li^+^. A relatively small first order structural change (displacive) of the LFP framework gives a two-phase separation over most of the solid solution range 0 < x < 1 for LixFePO_4_ - as seen in the flat voltage - charge curve. A reversible capacity of *ca*. 160 mAh g^−1^ was delivered by the C-coated LFP.

It has been reported elsewhere^25^ that two major limiting processes can explain why some Li^+^ cannot be fully extracted from the ordered LFP olivine structure, resulting in steady capacity fade:

1. Limited Li^+^ phase boundary diffusion – the one-dimensional channels in LFP impose structural constraint. In this instance Li^+^ diffusion can be interrupted by ionic disorder, foreign phases or stacking faults. And it is this interruption that impedes the motion of a LiFePO_4_/FePO_4_ phase boundary, effectively reducing the capability of a portion of the cathode from reversible intercalation activity.
2. Electron conductivity within the cathode – if electrons cannot transfer rapidly, at the specified C rate, then such mobility constraints will limit the Li^+^ insertion/extraction and this will result in diminishing capacity.

**Figure 8:**
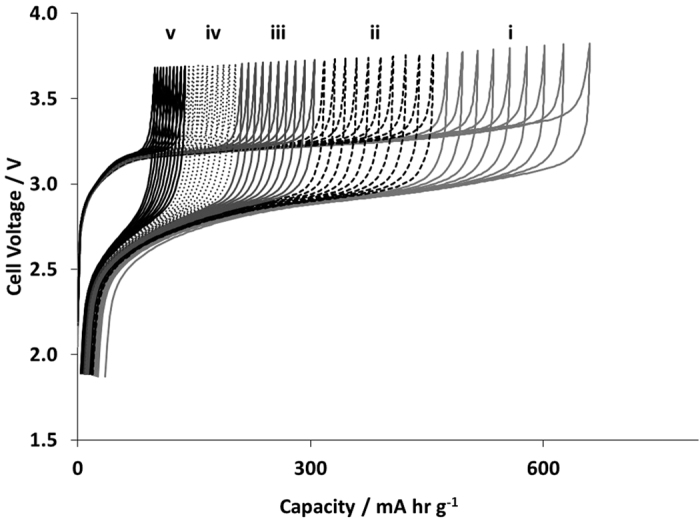
Extended voltage profiles characterising the cell voltage vs. capacity for successive cycle numbers performed within the three electrode cell.

Figure 8 extends the voltage-capacity plot from Fig. 7 to cycles 2–50 in the three electrode cell. It is evident that the capacity fade is quite pronounced over a relatively short cycle life. For the Si-FLG anode, the capacity was reset to zero at the start of every cycle, rather than continuing from the value at end of the previous cycle. The voltage alignment is consistent with the degree of lithiation. This implies that there is irreversible loss of active lithium e.g. in SEI growth, rather than lithium being kinetically trapped within the silicon structure.

### Differential Capacity Evaluation in Half Cells

Differential capacity analysis (dQ/dV) was used to monitor phase transitions and capacity fade, as a function of cycle number. It is a valuable non-destructive tool when attempting to establish degradation mechanisms in a cell. dQ/dV was calculated from the silicon and C/V-LFP electrodes data in half cells against lithium, and in full cells against each other. Analysis using dQ/dV has been used on a number of lithium ion battery chemistries^32^,^33^,^34^. For dQ/dV vs. voltage plots, the peaks corresponded to specific processes relating to phase equilibria as a function of changing voltages.

#### Si electrode dQ/dV Evaluation

**Figure 9:**
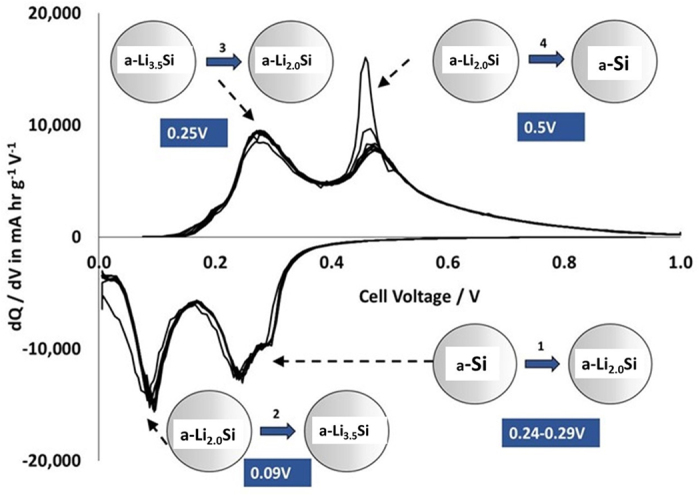
Differential capacity analysis (dQ/dV): the first derivative of the galvanostatic curve over cycles 2–10. Each peak is accompanied by diagrams explaining the structural transformations that are taking place within the anode as a function of the highlighted voltages.

Figure 9 shows the differential capacity (dQ/dV) plots for cycle numbers two to ten in the potential range 0–1.0 V to a capacity of 3000 mAh g^−1^, showing information about structural transformations during lithiation and delithiation for Si-FLG anodes vs. Li/Li^+^ in a half-cell. The profiles showed characteristic silicon lithiation and delithiation processes. The first cycle is completely different, being dominated by the transformation of crystalline Si (c-Si) to amorphous Si (a-Si). On subsequent cycles, the following peaks were observed:-

1. The first lithiation process between 0.25–0.3 V appeared as quite a broad doublet peak that could indicate two processes, likely to be the gradual lithiation of the a-Si lattice to form a-Li_2.0_Si. According to Grey *et al.* this phase is still composed of extended Si networks and large Si-Si clusters^35^.
2. The second lithiation process occurring in the range 0.09 to 0.10 V, and corresponded to the formation of amorphous-Li_3_._5_Si. This process involves the further breaking of Si-Si bonds to form small Si clusters and eventually isolated Si atoms. On cycle 2 only, there was a small peak at *ca*. 50 mV, which has been reported to correspond to the formation of c-Li_3.75_Si from a-Li_x_Si^36^,^37^,^38^.
3. The delithiation at 0.27 V corresponded to the conversion of Li_3.5_Si back to a-Li~_2.0_Si.
4. There are two possible reactions that could have occurred in the delithiation peak at 0.45 V. One involves the a-Li~2.0Si material. However, if any of the c-Li_3.75_Si phase had been formed, it would have also delithiated at this voltage. The original crystalline c-Si structure is never re-established.

With reference to the graphene content, dQ/dV peaks have been reported elsewhere at 0.07 V and consistent with the Li^+^ insertion into graphene^39^. Cycle two in Fig. 9 shows a lithiation peak at 0.07 V, but this was not seen in subsequent cycles. It has been reported elsewhere^39^ for a silicon: graphene 1:1 composite, that the graphene contribution to the lithiation process is seen in peaks at 1.0 and 0.05 V. Whilst there were consistent peaks around 1.0 V we did not see any at 0.05 V; the graphene content in this study is much lower than the silicon content, and hence, silicon represents the dominant peak areas. As the number of cycles increased to *ca*. 170, there was a gradual reduction in the peak intensity during delithiation, as shown in Supplementary Figure S4. Also, there was a shift in the lithiation peak voltages towards lower values, suggesting increasing resistance with higher cycle number. The increase in the cell resistance is attributable to a number of phenomena, including growth of resistive films on the active particle surfaces and loss of electrolyte from decomposition reactions. Both these phenomena can affect silicon anodes significantly.

## Conclusions

Electrochemical testing in half cells data for 5 at. % V-doped LFP showed interesting rate behaviour at reasonably high C-rates, with lighter coatings approaching maximum theoretical capacity. However, the cells also exhibited relatively low coulombic efficiency, and this contributed to capacity fade and limited capacity retention during cycling in full cells. Incorporating graphene in the V-LFP cathode showed no obvious benefit to rate performance in these experiments, but more investigation is needed into causes of the low coulombic efficiency of the cathode dominating capacity fade. Half-cell test results for Si-FLG composites showed promising capacities of ~1800 mAh g^−1^ for 150 charge-discharge cycles. The presence of the graphene improves the capacity retention in silicon half cells.

In full cells, there are more pronounced performance deterioration effects, since there is no compensatory excess of lithium as exists in half-cells. Thus, diminishing levels of lithium, due to multiple irreversible loss mechanisms associated with the active materials, cannot be replenished continually to the point where the cell fails. The finite lithium availability in a full cell, coupled with SEI growth at the silicon anode and relatively low coulombic efficiency at both electrodes, resulted in reduced cycle life, and more rapid capacity fade than would be acceptable in a commercial cell.

The presence of the graphene is likely to improve both the electrically conductive pathways and also the mechanical properties of the composite silicon coating, resulting in greater resistance to physical degradation, despite the expansive stresses generated by the silicon particles. However, more work is needed to investigate and quantify exactly what multi-layer graphene contributes to the physical and electrochemical performance properties of electrodes.

## Additional Information

**How to cite this article**: Loveridge, M. J. *et al.* Towards High Capacity Li-Ion Batteries Based on Silicon-Graphene Composite Anodes and Sub-micron V-doped LiFePO_4_ Cathodes. *Sci. Rep.* **6**, 37787; doi: 10.1038/srep37787 (2016).

**Publisher's note:** Springer Nature remains neutral with regard to jurisdictional claims in published maps and institutional affiliations.

## Supplementary Material

Supplementary Information
