# Supplementary material for: Towards High Capacity Li-ion Batteries Based on Silicon-Graphene Composite Anodes and Sub-micron V-doped LiFePO4 Cathodes
Source: Sci Rep. 2016 Nov 29;6:37787. doi: 10.1038/srep37787 (PMC5127186; doi:10.1038/srep37787)
Supplement: Supplementary Information [file srep37787-s1.pdf]

Towards High Capacity Li-ion Batteries Based on Silicon-Graphene Composite Anodes and Sub-micron V-doped $LiFePO_4$ Cathodes

M. J. Loveridge [a]*, M.J. Lain [a], I. Johnson [b], A. Roberts [a], S. D. Beattie [a], R. Dashwood, J. A. Darr [b] and R. Bhagat [a]

[a] Warwick University, Coventry, UK, CV4 7AL. [b] University College, London WC1E 6BT

* Corresponding author. Correspondence to m.loveridge@warwick.ac.uk

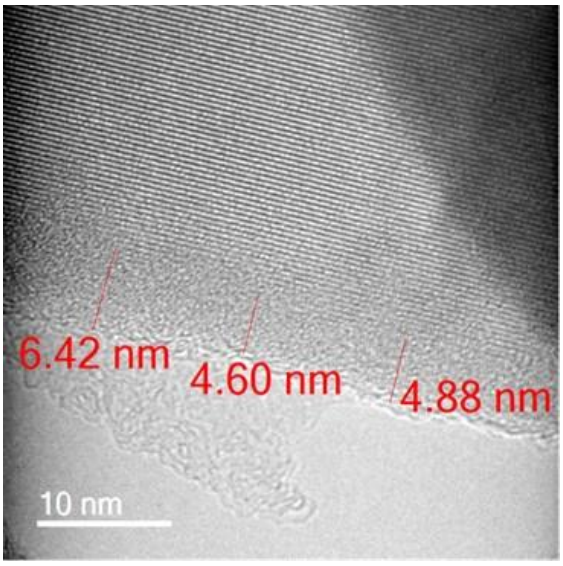

S1. Transmission Electron Micrograph (TEM) of the edge of a V-doped $LiFePO_4$ particle and is highlighting the uniformity of the thickness of the pyrolyzed carbon coating (introduced to improve the conductivity of the particles surfaces).

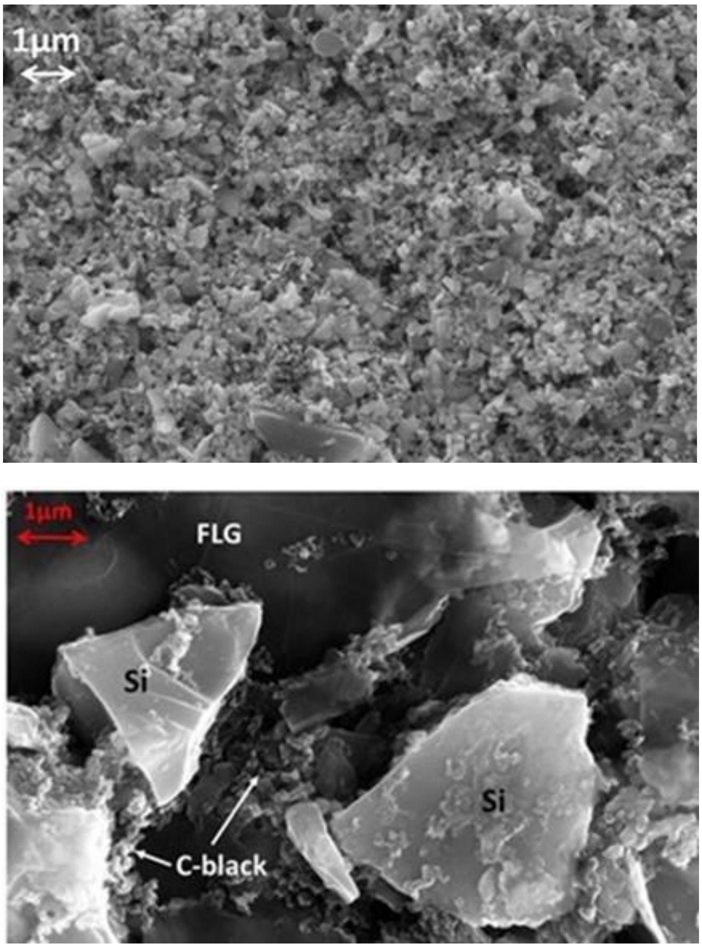

S2. Electrode microstructures with SEM images **a.** V-LFP cathode coating. **b.** Si-FLG anode coating. The FLG is seen repeatedly between silicon particles to generate long-range electrically conductive networks, with smaller carbon black particles supporting short-range conductivity.

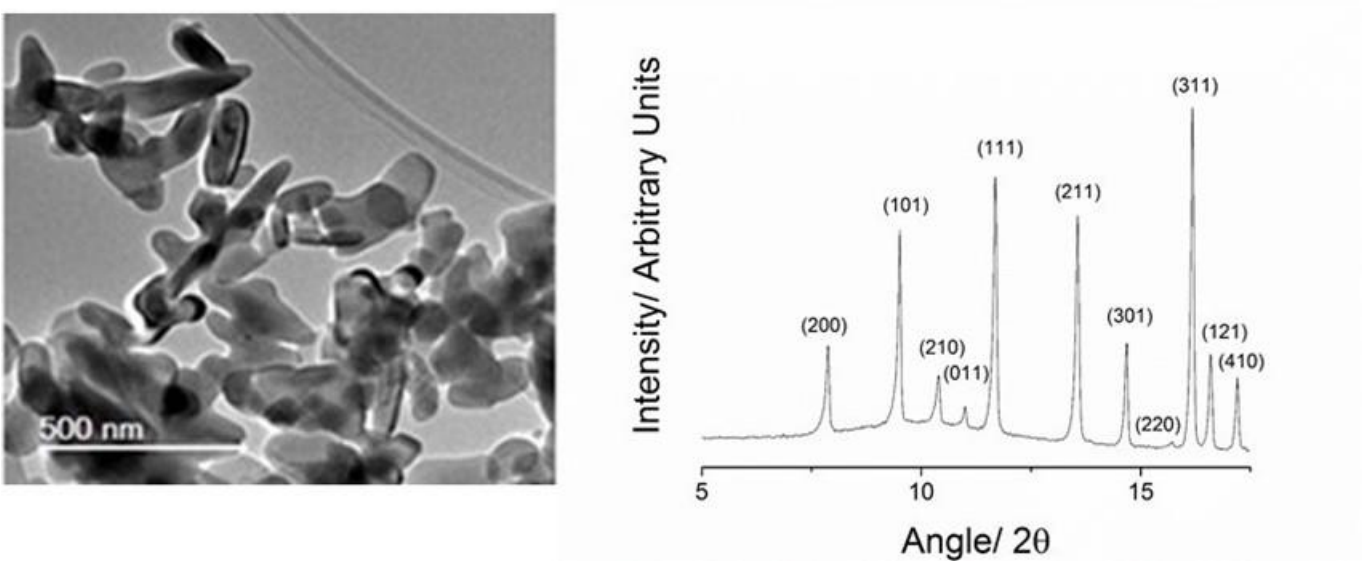

S3. Low and high magnification TEM of the V-LFP cathode particles showing a consistent particle size and aspect ratio. The X-ray diffractogram on the right shows the well-defined crystalline phases common to the V-LFP material and confirms that there are no impurity phases present.

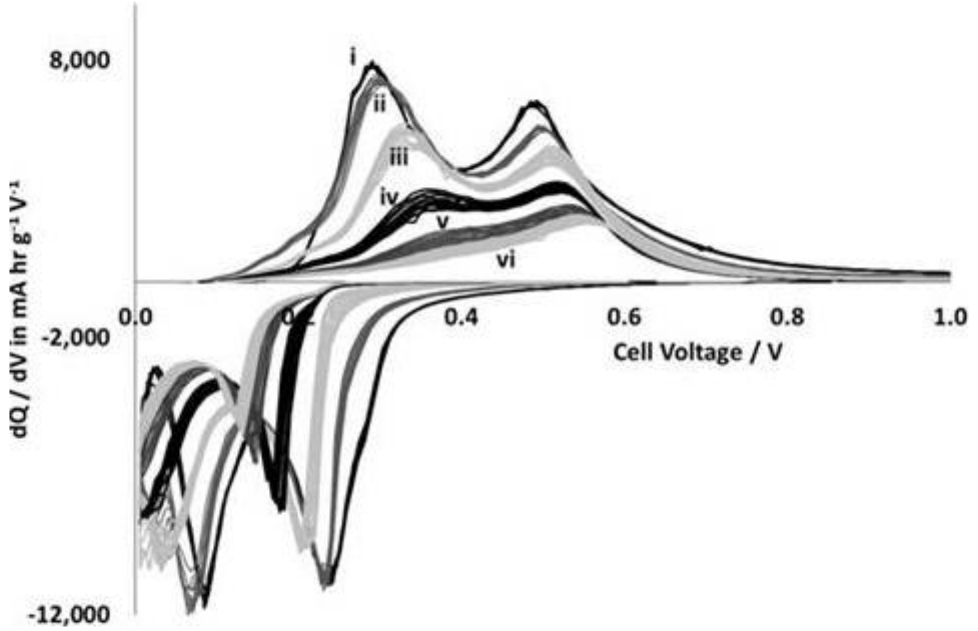

S4. Differential capacity plot (dQ/dV) to cycle 170 and is an extension of the dQ/dV plot in Figure 9 to characterise peak magnitudes and shifts as a function of cell voltage.

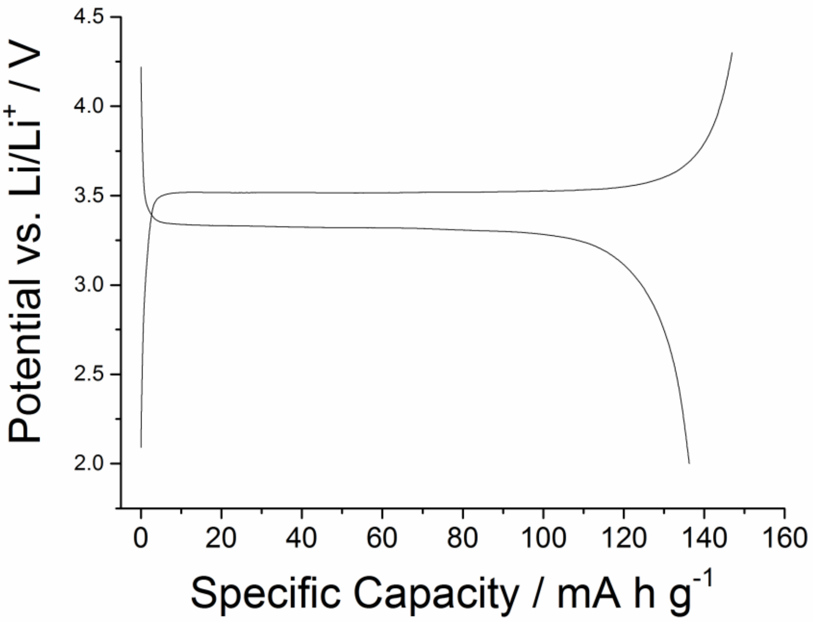

S5. Charge-discharge curves in a Potential (V) vs. Specific Capacity (mAh g^{-1}) plot to determine the maximum capacity achievable with the V-LFP material.
